# Effective Targeting of TAG72^+^ Peritoneal Ovarian Tumors via Regional Delivery of CAR-Engineered T Cells

**DOI:** 10.3389/fimmu.2018.02268

**Published:** 2018-11-19

**Authors:** John P. Murad, Anna K. Kozlowska, Hee Jun Lee, Maya Ramamurthy, Wen-Chung Chang, Paul Yazaki, David Colcher, John Shively, Mihaela Cristea, Stephen J. Forman, Saul J. Priceman

**Affiliations:** ^1^Department of Hematology and Hematopoietic Cell Transplantation, City of Hope, Duarte, CA, United States; ^2^Irell and Manella Graduate School of Biological Sciences, City of Hope, Duarte, CA, United States; ^3^Chair of Medical Biotechnology, Poznan University Medical Sciences, Poznań, Poland; ^4^Department of Molecular Imaging & Therapy, Diabetes Metabolism Research Institute of City of Hope, Duarte, CA, United States; ^5^Department of Medical Oncology & Therapeutics Research, City of Hope, Duarte, CA, United States; ^6^Department of Immuno-Oncology, Beckman Research Institute of City of Hope, Duarte, CA, United States

**Keywords:** chimeric antigen receptor, ovarian cancer, regional intraperitoneal delivery, TAG72, tumor-associated glycoproteins, adoptive cellular immunotherapy, STn, sialyl-Tn

## Abstract

Impressive clinical efficacy of chimeric antigen receptor (CAR)-engineered T cell therapy for hematological malignancies have prompted significant efforts in achieving similar responses in solid tumors. The lack of truly restricted and uniform expression of tumor-associated antigens, as well as limited T cell persistence and/or tumor trafficking pose major challenges for successful translation of CAR T cell therapy in solid tumors. Recent studies have demonstrated that aberrantly glycosylated cell surface proteins on tumor cells are amenable CAR targets. Tumor-associated glycoprotein 72 (TAG72) antigen is the sialyl-Tn found on multiple O-glycoproteins expressed at high levels on the surface of several cancer types, including ovarian cancer. Here, we developed a humanized TAG72-specific CAR containing a 4-1BB intracellular co-stimulatory signaling domain (TAG72-BBζ). TAG72-BBζ CAR T cells showed potent antigen-dependent cytotoxicity and cytokine production against multiple TAG72^+^ ovarian cancer cell lines and patient-derived ovarian cancer ascites. Using *in vivo* xenograft models of peritoneal ovarian tumors, regional intraperitoneal delivery of TAG72-BBζ CAR T cells significantly reduced tumor growth, extended overall survival of mice, and was further improved with repeat infusions of CAR T cells. However, reduced TAG72 expression was observed in early recurring tumors, which coincided with a lack of T cell persistence. Taken together, we demonstrate efficacy with TAG72-CAR T cells in ovarian cancer, warranting further investigations as a CAR T cell therapeutic strategy for this disease.

## Introduction

Chimeric antigen receptor (CAR)-engineered T cell therapy in patients with CD19+ B-cell malignancies have demonstrated impressive clinical responses (1, 2), which recently resulted in two landmark FDA approvals for patients with leukemia and lymphoma. These studies have shown that CAR T cells can be optimized to induce durable and complete responses in cancer patients, even under conditions of highly refractory disease. Major obstacles in developing effective CAR T cell therapies for solid cancers are avoiding off-tumor on-target toxicity due to the lack of truly restricted tumor antigens, as well as achieving durable responses that are limited by T cell persistence and tumor trafficking (3, 4). To date, the majority of tumor antigens for directing specificity of CAR T cells have targeted over-expressed proteins, including but not limited to mesothelin, PSMA, PSCA, HER2/neu, EGFR, and IL13Rα2 (3, 5). While the field is still evolving, clinical efficacy of CAR T cells targeting these proteins in solid tumors has been somewhat limited (6), and identification of additional targets as well as addressing limited T cell durability is critically important to the successful translation of CAR T cell therapies.

Aberrantly glycosylated cell surface proteins have long been implicated in tumor development, and have unique glycoprotein signatures that are attractive targets for immunotherapy, including CAR T cells (7, 8). Multiple cancer types including colon, breast, pancreas, and ovarian, are known to over-express aberrantly glycosylated proteins, including the mucins MUC16 and MUC1, and the tumor-associated glycoprotein 72 antigen (TAG72) (9), that differentiate them from normal epithelia. TAG72 is the truncated sialyl Tn (STn) O-glycan carbohydrate hapten located on multiple cell surface O-glycoproteins (10). High expression of TAG72, MUC1, and MUC16 has been shown in ovarian cancer patient tissue samples, with nearly 100% of ovarian cancers identified with simultaneous staining of the three antigens (11). Importantly, approximately 90% of epithelial ovarian cancers are TAG72 positive, indicating its abundance across multiple histological subtypes of ovarian cancer (11).

Several monoclonal antibodies that primarily target the tumor-associated STn have been developed, including the well-studied clone, CC49 (12). CC49 has been subsequently utilized in multiple pre-clinical and clinical investigations using diagnostic imaging and radiotherapy (13–16) and also involved in multiple attempts of antibody humanization (17–20). An early clinical trial of a first-generation CAR T cell targeting TAG72 in colorectal cancer patients demonstrated safety, but with limited anti-tumor responses, likely attributed to the limited T cell persistence and/or anti-idiotype responses from inadequate scFv humanization (21). Given the optimization of CAR T cells in recent years, and the incorporation of intracellular co-stimulatory signaling domains in second-generation CARs that has greatly improved anti-tumor activity, cytokine production and T cell persistence, an evaluation of second-generation CAR T cells targeting TAG72 warrants further investigation.

Here, we describe the generation and anti-tumor efficacy of a second-generation CAR T cell with a humanized anti-human TAG-72 scFv antigen-binding domain and a 4-1BB intracellular co-stimulatory signaling domain (TAG72-BBζ). *In vitro*, TAG72-BBζ CAR T cells demonstrate potent antigen-dependent cytotoxicity against multiple TAG72-expressing human ovarian cancer cell lines and epithelial cells derived from patient ovarian cancer ascites. Furthermore, using *in vivo* peritoneal ovarian tumor models, we show that regional intraperitoneal delivery of TAG72-BBζ CAR T cells eliminate antigen-positive disease and extends overall survival of mice, while intravenous CAR T cell delivery was ineffective in controlling disease. We also demonstrate that repeat regional infusions of CAR T cells promote more durable control of disease compared to single treatment. However, reduced TAG72 expression was observed in early recurring tumors, which coincided with a lack of T cell persistence in our models. Interestingly, late recurring tumors showed re-expression of TAG72, which will require additional mechanistic investigations. These preclinical findings support TAG72-BBζ CAR T cells as a viable therapeutic option for ovarian cancers, and also highlight its broader application for multiple TAG72-expressing solid cancers.

## Materials and methods

### Cell lines

The epithelial ovarian cancer line OVCAR-3 (herein referred to as OVCAR3, ATCC HTB-161) was cultured in RPMI-1640 (Lonza) containing 20% fetal bovine serum (FBS, Hyclone) and 1X antibiotic-antimycotic (1X AA, Gibco) (complete RPMI). The epithelial ovarian cancer line derived from metastatic ascites OV-90 (herein referred to as OV90, CRL-11732) was cultured in a 1:1 mixture of MCDB 105 medium (Sigma) and Medium 199 (Thermo) adjusted to pH of 7.0 with sodium hydroxide (Sigma) and final 20% FBS and 1X AA. The epithelial-endometroid ovarian cancer line COV362.4 (Sigma) was cultured in Dulbecco's Modified Eagles Medium (DMEM, Life Technologies) containing 10% FBS, 1X AA, 25 mM HEPES (Irvine Scientific), and 2 mM L-Glutamine (Fisher Scientific) (complete DMEM). The epithelial ovarian cancer line OVCAR-8 (herein referred to as OVCAR8) was a generous gift from Dr. Carlotta Glackin at City of Hope and was cultured in complete RPMI-1640. The epithelial ovarian cancer line SK-OV-3 (herein referred to as SKOV3, ATCC HTB-77) and the colon epithelial cancer line LS 174T (herein referred to as LS174T, ATCC CL-188) were cultured in complete DMEM. DU145-PSCA cells were described previously (22). All cells were cultured at 37°C with 5% CO_2_.

### DNA constructs and lentivirus production

Tumor cells were engineered to express enhanced green fluorescent protein and firefly luciferase (eGFP/*ffluc*) by transduction with epHIV7 lentivirus carrying the eGFP/*ffluc* fusion under the control of the EF1α promoter as described previously (22). The humanized scFv sequence used in the CAR construct was obtained from a monoclonal antibody clone huCC49 that targets TAG72 (17). The extracellular spacer domain included the 129-amino acid middle-length CH2-deleted version (ΔCH2) of the IgG4 Fc spacer (23). The intracellular co-stimulatory signaling domain contained was a 4-1BB with a CD4 transmembrane domain. The CD3ζ cytolytic domain was previously described (22). The CAR sequence was separated from a truncated CD19 gene (CD19t) by a T2A ribosomal skip sequence, and cloned in an epHIV7 lentiviral backbone under the control of the EF1α promoter. The PSCA-BBζ CAR construct was described previously (22).

Lentivirus was generated as previously described (22, 24). Briefly, 293T cells were transfected with packaging plasmid and CAR lentiviral backbone plasmid using a modified calcium phosphate method. Viral supernatants were collected after 3–4 days and treated with 2 mM magnesium and 25 U/mL Benzonase® endonuclease (EMD Millipore). Supernatants were concentrated via high-speed centrifugation and lentiviral pellets were resuspended in phosphate-buffered saline (PBS)-lactose solution (4 g lactose per 100 mL PBS), aliquoted and stored at −80°C. Lentiviral titers were quantified using HT1080 cells based on CD19t expression.

### T cell isolation, lentiviral transduction, and *ex vivo* expansion

Leukapheresis products were obtained from consented research participants (healthy donors) under protocols approved by the City of Hope Internal Review Board (IRB). On the day of leukapheresis, peripheral blood mononuclear cells (PBMC) were isolated by density gradient centrifugation over Ficoll-Paque (GE Healthcare) followed by multiple washes in PBS/EDTA (Miltenyi Biotec). Cells were rested overnight at room temperature (RT) on a rotator, and subsequently washed and resuspended in X-VIVO T cell medium (Lonza) containing 10% FBS (complete X-VIVO). Up to 5.0 × 10^9^ PBMC were incubated with anti-CD14 and anti-CD25 microbeads (Miltenyi Biotec) for 30 min at RT and magnetically depleted using the CliniMACS® system (Miltenyi Biotec) according to the manufacturer's protocol and these were termed depleted PBMCs (dPBMC). dPBMC were frozen in CryoStor® CS5 (StemCell Technologies) until further processing.

T cell activation and transduction was performed as described previously (22). Briefly, freshly thawed dPBMC were washed once and cultured in complete X-VIVO containing 100 U/mL recombinant human IL-2 (rhIL-2, Novartis Oncology) and 0.5 ng/mL recombinant human IL-15 (rhIL-15, CellGenix). For CAR lentiviral transduction, T cells were cultured with CD3/CD28 Dynabeads® (Life Technologies), protamine sulfate (APP Pharmaceuticals), cytokine mixture (as stated above), and desired lentivirus at a multiplicity or infection (MOI) of 1 the day following bead stimulation. Cells were then cultured in and replenished with fresh complete X-VIVO containing cytokines every 2–3 days. After 7 days, beads were magnetically removed, and cells were further expanded in complete X-VIVO containing cytokines to achieve desired cell yield. CAR T cells were positively selected for CD19t using the EasySep™ CD19 Positive Enrichment Kit I or II (StemCell Technologies) according to the manufacturer's protocol. Following further expansion, cells were frozen in CryoStor® CS5 prior to *in vitro* functional assays and *in vivo* tumor models. Purity and phenotype of CAR T cells were verified by flow cytometry.

### Flow cytometry

For flow cytometric analysis, cells were resuspended in FACS buffer (Hank's balanced salt solution without Ca^2+^, Mg^2+^, or phenol red (HBSS^−/−^, Life Technologies) containing 2% FBS and 1 × AA). Cells were incubated with primary antibodies for 30 min at 4°C in the dark. For secondary staining, cells were washed twice prior to 30 min incubation at 4°C in the dark with either Brilliant Violet 510 (BV510), fluorescein isothiocyanate (FITC), phycoerythrin (PE), peridinin chlorophyll protein complex (PerCP), PerCP-Cy5.5, PE-Cy7, allophycocyanin (APC), or APC-Cy7 (or APC-eFluor780)-conjugated antibodies. Antibodies against CD3 (BD Biosciences, Clone: SK7), CD4 (BD Biosciences, Clone: SK3), CD8 (BD Bosciences, Clone: SK1), CD14 (BD Biosciences, Clone: MΦP9), CD19 (BD Biosciences, Clone: SJ25C1), CD25 (BD Biosciences, Clone: 2A3), mouse CD45 (BioLegend, Clone: 30-F11), CD45 (BD Biosciences, Clone: 2D1), CD69 (BD Biosciences, Clone: L78), CD137 (BD Biosciences, Clone: 4B4-1), MUC1 (BioLegend, Clone 16A), MUC16 (Abcam, Clone X75 or EPSISR23), biotinylated Protein-L (GenScript USA) (25), TAG72 (Clone, muCC49), Donkey Anti-Rabbit Ig (Invitrogen), Goat Anti-Mouse Ig (BD Biosciences), and streptavidin (BD Biosciences) were used. Cell viability was determined using 4′, 6-diamidino-2-phenylindole (DAPI, Sigma). Flow cytometry was performed on a MACSQuant Analyzer 10 (Miltenyi Biotec), and the data was analyzed with FlowJo software (v10, TreeStar).

### *In vitro* tumor killing and T cell functional assays

For tumor killing assays, CAR T cells and tumor targets were co-cultured at indicated effector:tumor (E:T) ratios in complete X-VIVO in the absence of exogenous cytokines in 96-well plates for 24–72 h and analyzed by flow cytometry as described above. Tumor cells were plated overnight prior to addition of T cells. Tumor killing by CAR T cells was calculated by comparing CD45-negative DAPI-negative (viable) cell counts relative to that observed when targets were co-cultured with Mock (untransduced) T cells. For T cell activation assays, CAR T cells and tumor targets were co-cultured at the indicated E:T ratios in complete X-VIVO in the absence of exogenous cytokines in 96-well plates for the indicated time points and analyzed by flow cytometry for specific markers of T cell activation. Frozen, uncultured patient primary ovarian cancer ascites (OAS3, OAS4, and OAS7) were thawed and immediately evaluated in T cell functional assays. A ascites fluid from ovarian cancer patients was obtained from City of Hope National Medical Center (COH) surgical staff in a sterile vacuum container with approval from the COH Institutional Review Board (IRB) and Office of Human Subjects Protection. The COH IRB waived the need for written informed consent as all samples were de-identified and ascites was discard material as previously described (26).

For T cell activation assays on plate-bound antigen, purified soluble TAG72 antigen (BioRad) was plated in duplicate at indicated TAG72 units overnight at 4°C in 1X PBS in 96-well flat bottom high-affinity plates (Corning). Using a Bradford protein assay, the 20,000 units/mL stock solution of soluble TAG72 antigen was determined to be approximately 1.234 mg/mL of total protein. A total of 10^4^ TAG72-BBζ CAR T cells were then added in a fixed volume of 100 μL to each well and incubated for indicated times prior to collection of cells for analysis of activation markers (CD69, CD137) by flow cytometry. Supernatants were also collected for analysis of cytokine production.

### Elisa cytokine assays

Supernatants from tumor killing assays or CAR T cell activation assays on plate-bound TAG72 antigen were collected at indicated times and frozen at −20°C for further use. Supernatants were then analyzed for secreted human IFNγ and IL-2 according to the Human IFNγ and IL-2 ELISA Ready-SET-GO!^®;^ ELISA kit manufacturer's protocol, respectively. Plates were read at 450 nm using a Wallac Victor3 1420 Counter (Perkin-Elmer) and the Wallac 1420 Workstation software.

### *In vivo* tumor studies

All animal experiments were performed under protocols approved by the City of Hope Institutional Animal Care and Use Committee. For *in vivo* tumor studies, OVCAR3 and OV90 cells (5.0 × 10^6^) were prepared in a final volume of 500 μl HBSS^−/−^ and engrafted in 6–8 weeks old female NSG mice by intraperitoneal (i.p.) injection. Tumor growth was monitored at least once a week via biophotonic imaging (Xenogen, LagoX) and flux signals were analyzed with Living Image software (Xenogen). For imaging, mice were i.p. injected with 150 μL D-luciferin potassium salt (Perkin Elmer) suspended in PBS at 4.29 mg/mouse. Once flux signals reached desired levels, day 8 for OV90 and day 14 for OVCAR3, T cells were prepared in 1X PBS, and mice were treated with 500 μL i.p. or 200 μL intravenous (i.v.) injection of 5.0 x 10^6^ Mock or TAG72-BBζ CAR T cells. In the OV90 tumor model, we tested the impact of repeat treatment with i.p. TAG72-BBζ CAR T cells starting at day 8, followed by treatments at additional indicated days post-tumor engraftment. Humane endpoints were used in determining survival. Mice were euthanized upon signs of distress such as a distended belly due to ascites, labored or difficulty breathing, apparent weight loss, impaired mobility, or evidence of being moribund. At pre-determined time points or at moribund status, mice were euthanized and tissues and/or ascites fluid were harvested and processed for flow cytometry and immunohistochemistry as described below.

Peripheral blood was collected from isoflurane-anesthetized mice by retro-orbital (RO) bleed through heparinized capillary tubes (Chase Scientific) into polystyrene tubes containing a heparin/PBS solution (1000 units/mL, Sagent Pharmaceuticals). Volume of each RO blood draw (approximately 120 μL/mouse) was recorded for cell quantification per μL blood. Red blood cells (RBCs) were lysed with 1X Red Cell Lysis Buffer (Sigma) according to the manufacturer's protocol and then washed, stained, and analyzed by flow cytometry as described above. Cells from i.p. ascites fluid was collected from euthanized mice by injecting 5 mL cold 1X PBS into the i.p. cavity, which was drawn up via syringe and stored on ice until further processing. RBC-depleted ascites was washed, stained, and analyzed by flow cytometry for tumor-associated glycoprotein expression and CAR T cells using antibodies and methods described above.

### Immunohistochemistry

Tumor tissue was fixed for up to 3 days in 4% paraformaldehyde (4% PFA, Boston BioProducts) and stored in 70% ethanol until further processing. Immunohistochemistry was performed by the Pathology Core at City of Hope. Briefly, paraffin-embedded sections (10 μm) were stained with hematoxylin & eosin (H&E, Sigma-Aldrich), mouse anti-human CD3 (DAKO), mouse anti-human TAG72 (AB16838, Abcam), rabbit anti-human MUC1 (AB45167, Abcam), MUC16 (AB1107, Abcam). Images were obtained using the Nanozoomer 2.0HT digital slide scanner and the associated NDP.view2 software (Hamamatzu).

### Statistical analysis

Data are presented as mean ± SEM, unless otherwise stated. Statistical comparisons between groups were performed using the unpaired two-tailed Student's *t*-test to calculate *p*-value, unless otherwise stated. ^*^p < 0.05, ^**^p < 0.01, ^***^p < 0.001; NS, not significant.

## Results

### TAG72-CAR T cells containing a 4-1BB intracellular co-stimulatory domain demonstrate *in vitro* activation against purified TAG72

**Figure 1:**
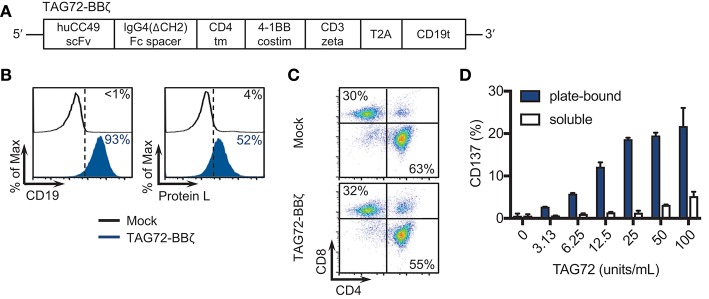
TAG72-specific CAR T cells containing a 4-1BB intracellular co-stimulatory domain. **(A)** Diagram of the lentiviral expression cassette with TAG72-CARs containing the humanized scFv (CC49 clone) targeting TAG72, with a 129 amino acid modified human IgG4 Fc linker (void of the CH2 domain, ΔCH2), a CD4 transmembrane domain, a cytoplasmic 4-1BB costimulatory domain, and a cytolytic CD3ζ domain. A truncated non-signaling CD19 (CD19t), separated from the CAR sequence by a T2A ribosomal skip sequence, was expressed for identifying lentivirally transduced T cells. **(B)** Mock (untransduced) and TAG72-BBζ CAR T cells were evaluated by flow cytometry for CD19t expression to detect lentiviral transduction of CARs (left) or Protein L to detect the scFv (right). **(C)** CD4 and CD8 expression in Mock (top) and TAG72-BBζ CAR T cells (bottom). **(D)** Activation (expression of CD137) was assessed by flow cytometry with *in vitro* stimulated CAR T cells against soluble or plate-bound purified TAG72 antigen for 24 h at indicated protein amounts.

Our first goal was to develop a second-generation TAG72-BBζ CAR construct containing the humanized scFv CC49, the human IgG4 Fc extracellular spacer lacking a CH2 domain (ΔCH2), the CD4 transmembrane domain, the 4-1BB intracellular co-stimulatory domain, and the CD3ζ cytolytic domain followed by a truncated CD19 (CD19t) for cell tracking (Figure 1A). We selected this CAR construct based on our recent preclinical investigations demonstrating potent anti-tumor activity of 4-1BB-containing CARs for solid tumors (22, 24, 27). TAG72-BBζ CAR lentivirus was used to transduce human healthy donor-derived peripheral blood mononuclear cells depleted of CD14+ and CD25+ cells (dPBMC), as previously described (22). TAG72-BBζ CAR T cells were enriched during the manufacturing process (based on CD19t+ selection) and were stably expressed on the surface of T cells (Figure 1B). CAR T cells expanded *ex vivo* with similar kinetics and comparable CD4:CD8 ratios to Mock (untransduced) T cells (data not shown and Figure 1C). Importantly, and as a first measure of CAR T cell activation against TAG72, we demonstrated dose-dependent CD137 expression on the surface of TAG72-BBζ CAR T cells when cultured with plate-bound, but not soluble, purified TAG72 (Figure 1D). Similar dose-dependent induction of cell-surface CD69 expression and IFNγ release was observed with plate-bound TAG72 (Supplemental Figures 1A,B).

### TAG72-BBζ CAR T cells effectively target ovarian cancer cells *in vitro*

**Figure 2:**
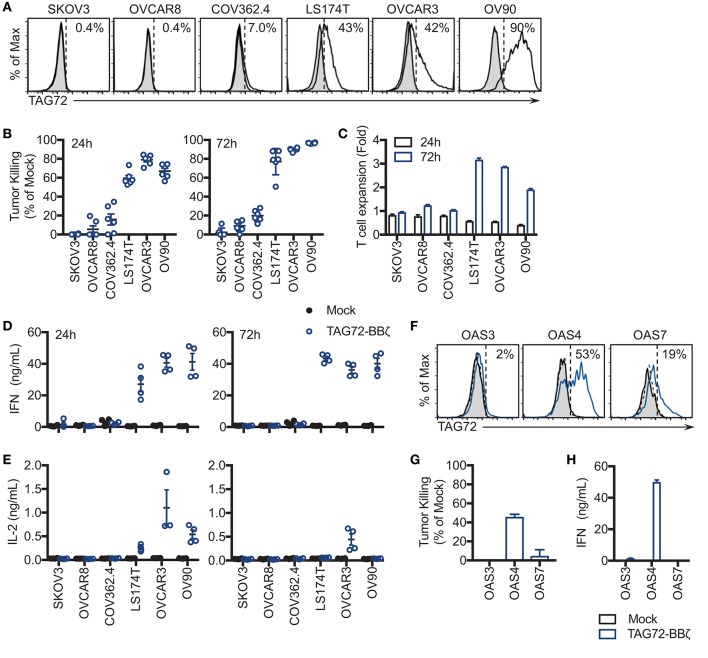
TAG72-BBζ CAR T cells show antigen-dependent cytokine production and tumor killing *in vitro*. **(A)** Flow cytometric analysis of TAG72 surface expression on multiple ovarian and colorectal (LS174T) cancer cell lines. **(B)** Quantification of tumor killing by TAG72-BBζ CAR T cells relative to Mock at an E:T ratio of 1:1, following a 24 and 72 h co-culture with antigen-positive and -negative tumor targets as described in Materials and Methods. **(C)** TAG72-BBζ CAR T cell expansion at 24 and 72 h following co-culture with indicated tumor targets at an E:T ratio of 1:1. **(D,E)** IFNγ and IL-2 levels in supernatant quantified by ELISA from Mock or TAG72-BBζ CAR T cells following a 24 and 72 h co-culture with indicated tumor targets at an E:T ratio of 1:1. **(F)** Flow cytometric analysis of TAG72 surface expression on primary human ovarian cancer cells harvested from patient ascites (OAS) after 72 h in culture. **(G)** Quantification of tumor killing and **(H)** IFNγ production by TAG72-BBζ CAR T cells relative to Mock following a 72 h co-culture with freshly thawed OAS cells at an E:T ratio of 1:1.

We next sought to evaluate cell-surface TAG72 expression on human ovarian cancer cell lines, including SKOV3, OVCAR8, COV362.4, OVCAR3, OV90, as well as the TAG72+ colon cancer line, LS174T. Prior studies have demonstrated expression of TAG72 by immunohistochemistry of ovarian tumor patient samples and by western blotting of human ovarian cancer cell lines (11, 28). By flow cytometry, TAG72 was expressed on OVCAR3 cells (approximately 42%) and to a greater extent on OV90 cells (approximately 90%), with very low levels detected on COV362.4 cells (Figure 2A). TAG72 was absent on SKOV3 and OVCAR8 cells. Immunofluorescence staining of tumor cells confirmed TAG72 expression and cellular localization on the cell surface as well as intracellularly (data not shown). Importantly, we observed higher expression of TAG72 on OVCAR3 and OV90 cells harvested from the ascites of tumor-bearing animals as compared to *in vitro* cultured cells (Supplemental Figure 2).

To assess antigen-dependent activity of our TAG72-BBζ CAR T cells, we performed co-cultured assays with TAG72-positive and -negative ovarian tumor targets at an E:T ratio between 1:1 and 1:2 to determine their killing potential. After 24 h, antigen-specific T cell-mediated killing activity was evident with TAG72-BBζ CAR T cells relative to Mock T cells (Figure 2B). Amongst TAG72-expressing targets, an average of 59% LS174T, 79% OVCAR3, and 67% OV90 cells were killed. After 72 h, killing of the same tumor lines increased to 77, 90, and 97%, respectively. TAG72-BBζ CAR T cells showed minimal killing of TAG72-negative or low expressing SKOV3, OVCAR8, and COV362.4 cells. We further demonstrated the specificity of our TAG72-CAR T cells using a previously described CAR targeting prostate stem cell antigen (PSCA) with the same CAR design (22) (Supplemental Figure 3). At 72 h, we observed TAG72-BBζ CAR T cell expansion (2–3 fold) against TAG72-positive tumor cells (Figure 2C). Similar tumor killing was observed at lower E:T ratios of 1:10 (data not shown), demonstrating the potent killing ability of TAG72-BBζ CAR T cells. TAG72 is shed from tumor cells in a soluble form (29), which we showed minimally impacted the tumor killing ability of TAG72-BBζ CAR T cells (Supplemental Figure 4). We then evaluated cytokine production from CAR T cells as an additional measure of T cell activity. IFNγ and IL-2 cytokine production was observed only when TAG72-BBζ CAR T cells were co-cultured with antigen-positive tumor targets, OVCAR3, LS174T, and OV90 (Figures 2D,E). While IL-2 production peaked at early time points (24 h) and was detectable only against OVCAR3 at later time points (72 h), IFNγ levels were more sustained over 72 h.

### TAG72-BBζ CAR T cells target TAG72-positive cells from ovarian cancer ascites *in vitro*

To further confirm TAG72 as an ovarian cancer CAR target and the anti-tumor activity of our TAG72-BBζ CAR T cells, we performed *in vitro* assays utilizing human ovarian cancer ascites from three patients (OAS3, OAS4, OAS7). Freshly thawed ascites from OAS3, OAS4, and OAS7 expressed 62, 80, and 67% TAG72, respectively, by flow cytometry (data not shown), but after 72 h in culture, was reduced to 2, 53, and 19%, respectively (Figure 2F), likely reflecting an influence of *ex vivo* culturing conditions on maintenance of TAG72 expression (30). We then evaluated the cytolytic activity of CAR T cells after 72 h of co-culture with freshly thawed patient primary ovarian cancer ascites, and showed potent and selective CAR-mediated killing of the TAG72-positive OAS4 and OAS7 cells, with no detectable anti-tumor activity against the TAG72-negative OAS3 cells (Figure 2G). TAG72-BBζ CAR T cells produced IFNγ and IL-2 against OAS4, but not OAS3 and OAS7 cells (Figure 2H, Supplemental Figure 5).

### Regional intraperitoneal delivery of TAG72-BBζ CAR T cells exhibits potent anti-tumor activity and extends survival in ovarian ascites-bearing mice

**Figure 3:**
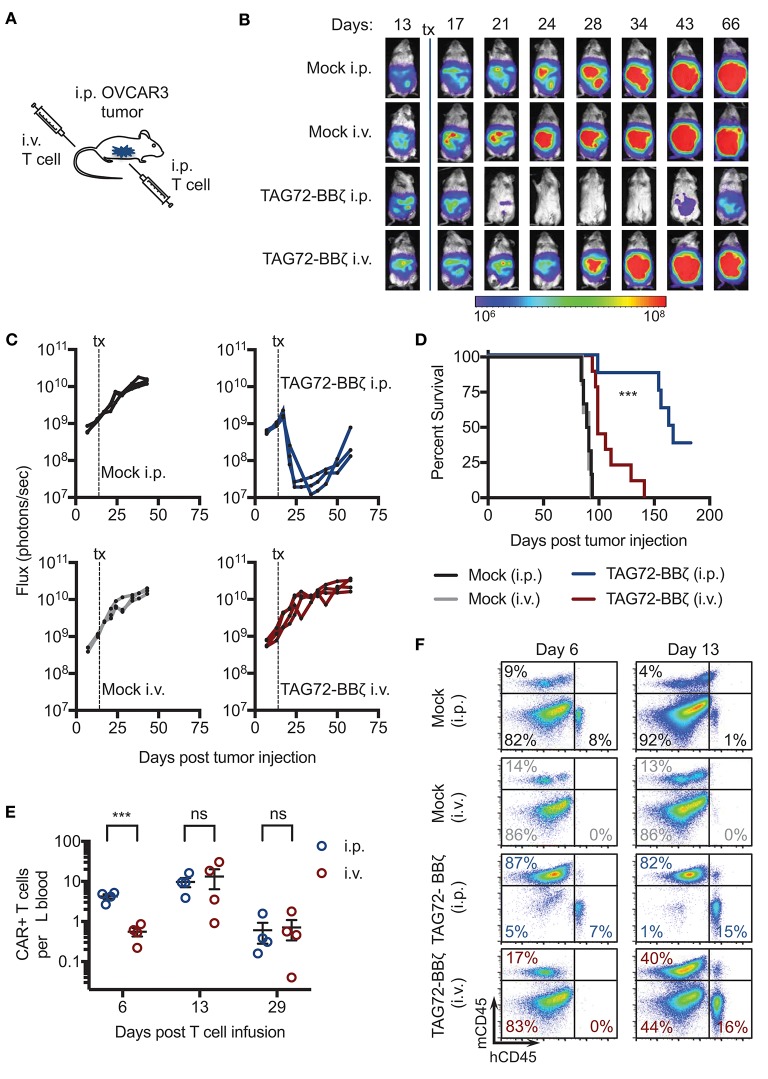
Regional intraperitoneal delivery of TAG72-BBζ CAR T cells significantly reduces tumor burden and extends overall survival of OVCAR3 tumor-bearing mice. **(A)** Schematic illustrating i.p. engraftment of 5.0 × 10^6^ OVCAR3(eGFP/*ffluc*) tumor cells in NSG mice, followed by either i.v. or i.p. delivery of 5.0 × 10^6^ Mock or TAG72-BBζ CAR T cells on day 14 post-tumor injection. **(B)** Representative bioluminescent flux imaging of mice treated i.v. or i.p. with Mock or TAG72-BBζ CAR T cells. **(C)** Quantification of flux (each mouse) from OVCAR3(eGFP/*ffluc*) tumor-bearing mice treated i.v. or i.p. with Mock or TAG72-BBζ CAR T cells. *N* = 3–4 per group. Data are representative of two independent experiments. **(D)** Kaplan–Meier survival for Mock and TAG72-BBζ CAR T cell treated mice. *N* ≥ 4 mice per group. Combined data from two independent experiments. **(E)** Quantification of TAG72-BBζ CAR T cells per μL blood at 6, 13, and 29 days post-treatment. *N* = 4 per group. **(F)** Representative flow cytometric analysis of the frequency of human CD45+ (hCD45) and mouse CD45+ (mCD45) cells in the i.p. cavity of tumor-bearing mice at day 6 and 13 post-treatment. Representative images from two independent experiments. ***p < 0.001.

To evaluate the therapeutic potential of our TAG72-BBζ CAR T cells *in vivo*, we first established TAG72+ OVCAR3 tumors in immune compromised NSG mice by intraperitoneal (i.p.) injection, to mimic peritoneal ovarian tumors observed in late-stage human disease. OVCAR3 cells were lentivirally transduced to express eGFP/*ffluc* to allow for tracking of tumor growth via non-invasive optical imaging. At 14 days post-tumor i.p. injection, mice were treated with Mock or TAG72-BBζ CAR T cells (5.0 × 10^6^) by systemic intravenous (i.v.) or regional i.p. delivery (Figure 3A). We observed rapid anti-tumor effects in mice treated with TAG72-BBζ CAR T cells via regional i.p. delivery, reaching a maximal anti-tumor response 1–2 weeks following treatment (Figures 3B,C). In comparison to regional delivery, i.v. delivery of TAG72-BBζ CAR T cells showed limited anti-tumor responses. Anti-tumor responses in mice were durable for 3–4 weeks, but ultimately tumor recurrences were observed in mice. Regional i.p. delivery of TAG72-BBζ CAR T cells significantly extended survival of mice, with limited benefits observed by i.v. delivery (Figure 3D).

To address potential differences observed between i.p. and i.v. therapy, we measured CAR T cells in the blood and ascites of mice. Strikingly, appreciable numbers of CAR T cells (huCD45+CD19t+) were found in the blood of mice 6 days post i.p. treatment, with more than 5-fold fewer CAR T cells in the blood of i.v. treated mice at the same time point (Figure 3E, Supplemental Figure 6). However, we observed equivalent numbers of CAR T cells in the blood of i.p. and i.v. treated mice at later time points, expanding from 1 to 2 weeks, with significant reductions at 4 weeks post-treatment. We then evaluated CAR T cell presence in the ascites of treated mice, and observed CAR T cells at the site of tumors at day 6 post i.p. treatment, with no detectable CAR T cells in i.v. treated mice at the same time point. However, at day 13 post-treatment, similar levels of CAR T cells were observed in mice treated i.v. and i.p. (Figure 3F). These data suggest that CAR T cells eventually reached the tumor following i.v. delivery but with delayed kinetics compared with i.p. delivery, which was likely in part responsible for the lack of observed therapy by this route of administration. CD45-negative cells, likely majority being OVCAR3 tumor cells, were significantly depleted in i.p. TAG72-BBζ CAR T cell treated mice, but not i.p. or i.v. Mock T cell or i.v. TAG72-BBζ CAR T cell treated mice. These data support regional intraperitoneal delivery of TAG72-CAR T cells as an effective method of targeting peritoneal ovarian tumors in mice.

### Repeat treatment with TAG72-BBζ CAR T cells controls tumor more effectively

**Figure 4:**
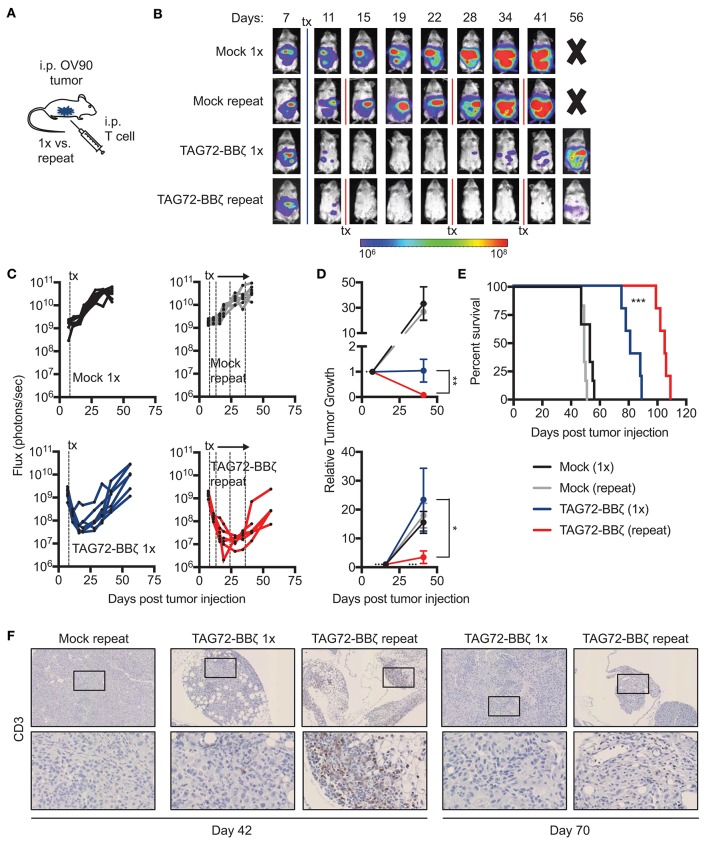
Repeat regional administration of TAG72-BBζ CAR T cells significantly reduces tumor burden and extends overall survival of OV90 tumor-bearing mice. **(A)** Schematic illustrating i.p. engraftment of 5.0 × 10^6^ OV90(eGFP/*ffluc*) tumor cells in NSG mice, followed by either single or repeat i.p. treatment with 5.0 × 10^6^ Mock or TAG72-BBζ CAR T cells on day 8 post-tumor infection. **(B)** Representative bioluminescent flux imaging of mice treated i.p. with a single or repeat treatment of Mock or TAG72-BBζ CAR T cells. **(C)** Quantification of flux (each mouse) from OV90(eGFP/*ffluc*) tumor-bearing mice with single or repeat i.p. treatment of Mock or TAG72-BBζ CAR T cells. **(D)** Top graph: relative tumor growth (flux) from day 7 (one day prior to start of treatment) to day 46 (end of the repeat treatment window). Bottom graph: relative tumor growth (flux) from day 16 (maximum treatment response) to day 46. Fluxes at day 7 or day 16 were normalized to “1” to reflect fold differences in tumor growth compared to day 46. Mann-Whitney test was performed to calculate *p*-values. **(E)** Kaplan-Meier survival for Mock and TAG72-BBζ CAR T cell treated mice. *N* ≥ 5 mice per group. **(F)** Histology of human CD3 cells in tumors harvested from single and repeat treated mice at days 42 and 70 post-tumor injection (top: 10X magnification, bottom: 40X magnification). All data are representative of two independent experiments. *p < 0.05, **p < 0.01, ***p < 0.001.

Based on our findings with TAG72-BBζ CAR T cells in OVCAR3-bearing mice, we next evaluated the OV90 i.p. model, with more uniform TAG72 expression *in vitro* compared with OVCAR3 (Figure 2A). We first confirmed effectiveness of regional CAR T cell delivery in this model and showed similarly to the OVCAR3 model, i.p., but not i.v. TAG72-BBζ CAR T cell treatment showed anti-tumor efficacy in the OV90 model (Supplemental Figure 7A). Overall survival was only delayed by approximately 25 days in this model with i.p. delivery of TAG72-BBζ CAR T cells (Supplemental Figure 7B), likely owing to the aggressive nature of this model. We therefore evaluated whether repeat TAG72-BBζ CAR T cell dosing compared with a single dose improves therapeutic responses (Figure 4A). Compared with a single dose of TAG72-BBζ CAR T cells, repeat dosing over the course of 1 month demonstrated more durable anti-tumor responses in the OV90 model (Figures 4B,C). When plotted as relative tumor growth kinetics, repeat dosing promoted more extensive tumor regression as well as more durable control of tumors compared with single dosing (Figure 4D).

In this study, the overall survival was extended significantly in mice that received repeat doses of TAG72-BBζ CAR T cells (55 day benefit) compared with a single dose (30 day benefit) (Figure 4E). Greater T cell numbers were observed in peritoneal tumors of mice with repeat treatment (Figure 4F). Importantly, however, we observed reduced numbers, expansion and persistence of CAR T cells in the blood of OV90-bearing mice, compared with the OVCAR3 model (Supplemental Figures 8A,B), suggesting that this more aggressive tumor model may also harbor suppressive mechanisms that hamper T cell function and overall CAR T cell efficacy. Collectively, these data demonstrate potent anti-tumor activity of TAG72-BBζ CAR T cells in ovarian cancer xenograft models, and also suggest that repeat dosing of regionally delivered CAR T cells may provide greater control of tumors compared with a single dose.

### Tumor recurrences following TAG72-CAR T cell therapy show antigen escape

**Figure 5:**
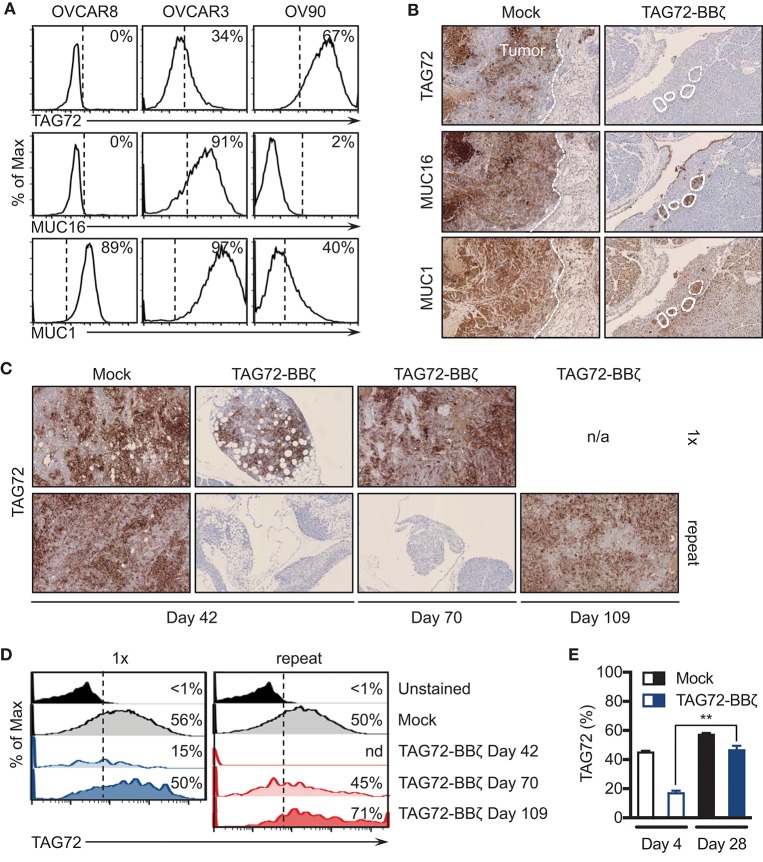
Tumor-associated glycoprotein antigen heterogeneity in ovarian cancer and CAR T cell-mediated antigen escape. **(A)** Flow cytometric analysis of TAG72, MUC16, and MUC1 surface expression on OVCAR8, OVCAR3, and OV90 human ovarian cancer cell lines. **(B)** Histology of TAG72, MUC16, and MUC1 expression in i.p. solid tumors harvested from Mock and TAG72-BBζ CAR T cell treated OVCAR3 tumor-bearing mice at day 99 post-treatment. 10X magnification. **(C)** Histology of TAG72 expression on solid tumors harvested from single and repeat treated OV90 tumor-bearing mice at day 42, 70, and 109 post-tumor injection. 10X magnification. **(D)** Flow cytometric analysis of TAG72 expression in OV90 tumor cells harvested from ascites at indicated time points from mice that received single or repeat i.p. treatment. **(E)** TAG72 expression on OVCAR3 cells at day 4 following co-culture with Mock or TAG72-BBζ CAR T cells (1:10 E:T ratio), and on tumor cells that grew out at day 28. **p < 0.01.

One of the major resistance mechanisms to CAR T cell therapy is the tumor antigen heterogeneity that exists in solid tumors that promotes eventual antigen loss or escape (4). While the loss of CAR T cells in our two models preceded tumor recurrences, we next evaluated expression of TAG72 in tumors from Mock and TAG72-BBζ CAR T cell treated mice at various time points pre- and post- therapy. Since TAG72, MUC1, and MUC16 have all been identified as potential targets in ovarian cancer, we first assessed expression of these cell surface antigens on TAG72-negative OVCAR8, and TAG72-positive OVCAR3 and OV90 cells. OVCAR8 appeared to only express low levels of MUC1, and was absent for TAG72 and MUC16, while OVCAR3 expressed all three antigens at varying levels, and OV90 showed low expression of MUC1 and was absent for MUC16 (Figure 5A). Therefore, we evaluated the expression of these antigens in OVCAR3 tumors from mice treated with Mock or TAG72-BBζ CAR T cells. At 12 weeks post T cell infusion, tumors from Mock-treated mice showed heterogeneous expression of TAG72 (similar to flow cytometric analysis of the cell line), MUC16, and MUC1 (Figure 5B). However, tumor recurrences at early time points from mice treated with TAG72-BBζ CAR T cells showed a dramatic reduction in TAG72 expression, while maintaining expression of MUC16 and MUC1. Similarly, repeat treatment of TAG72-BBζ CAR T cells in the OV90 tumor model also showed a reduction in TAG72 expression in early recurrent tumors following treatment (Figure 5C). Interestingly, the expression of TAG72 was detected at high levels in tumor recurrences at later time points, in solid tumors as well as in ascites (Figures 5C,D). We further confirmed this finding *in vitro*, showing that residual viable OVCAR3 tumor cells remaining after CAR T cell co-culture expressed lower TAG72, but showed typical TAG72 expression levels on tumor cells that grew out in the absence of CAR T cells (Figure 5E). Similar reductions in TAG72 expression were observed with OV90 cells (data not shown). These data suggest that antigen escape plays a key role in tumor recurrences following TAG72-BBζ CAR T cell therapy.

## Discussion

In this study, we evaluated a second-generation TAG72-specific CAR T cell with a 4-1BB intracellular co-stimulatory signaling domain in preclinical models of ovarian cancer. TAG72-CAR T cells demonstrated significant anti-tumor activity against peritoneal ovarian tumors when administered via regional intraperitoneal delivery. While we did not directly compare our CAR construct to first-generation TAG72-CARs, which have been previously published (21, 31, 32), substantial data in the field now show superiority of CARs containing co-stimulatory domains compared with first-generation CD3ζ-only CARs (3). Our studies also did not evaluate TAG72-CARs containing CD28 co-stimulation. However, our recent findings with PSCA- and HER2-directed CAR T cells show that while CD28-containing CAR T cells exhibit potent anti-tumor activity in solid tumors, undesirable increases in T cell exhaustion markers, limited persistence, and targeting of tumor cells that express very low levels of antigen may potentiate off-tumor toxicity, compared with 4-1BB-containing CARs (22, 24). Similar findings have been observed by other groups (33–35). In a recent publication detailing the use of TAG72-ζ CAR T cells in the context of colorectal cancer, systemic administration of CAR T cells was well tolerated in patients and demonstrated signs of transient on-target activity (21). However, limited anti-tumor responses in these patients was in part attributed to a lack of T cell persistence with a first-generation CAR construct lacking co-stimulation. In addition to recent *in vitro* work that highlight the potential of TAG72-directed CARs with co-stimulation (36), our current study demonstrates the anti-tumor activity of second-generation humanized TAG72-BBζ CAR T cells using clinically relevant *in vivo* models of ovarian cancer. While safety of targeting STn antigens (i.e., TAG72) with our CAR T cells was not addressed in the current study, the early clinical experience with first-generation TAG72-CAR T cells (21), along with recent studies using antibody-drug conjugates in non-human primates (37) provides some evidence of safety in targeting these antigens. Further clinical studies are required to evaluate the safety of these new second-generation TAG72-CAR T cells in patients.

Recent work by our group and others have suggested that regional administration of CAR T cells may improve CAR T cell therapeutic efficacy in several solid tumor models (24, 38–40). Our studies also demonstrate potent anti-tumor activity by regional intraperitoneal administration of TAG72-CAR T cells, compared to limited activity with intravenous delivery, using xenograft models of peritoneal ovarian tumors. In our models, i.v. administered CAR T cells did show trafficking to tumor sites at later time points, however, i.p. administered CAR T cells were observed at tumor sites early following treatment, likely driving more immediate anti-tumor responses, compared with systemic delivery of CAR T cells (Figure 3). Similar findings were observed previously with CAR T cells targeting peritoneal ovarian tumors (40). We believe that the delayed kinetics in T cell trafficking by i.v. delivery may have insufficiently controlled tumor burden compared with regional delivery, which may be overcome by increasing the dose of i.v. delivered CAR T cells in this model. However, the clinically feasible regional delivery of CAR T cells may provide immediate anti-tumor activity with improved overall therapeutic responses.

Antigen heterogeneity is a major obstacle to the successful translation of CAR T cell therapies for solid tumors. Expression analysis of MUC1, MUC16, and TAG72 on patient samples from various epithelial subtypes of ovarian cancer highlights antigen heterogeneity in this disease and demonstrates the aberrant expression pattern of cell-surface glycoproteins. Although we show antigen-specific targeting and extended survival of mice treated with our TAG72-CAR T cells using two human xenograft peritoneal ovarian tumor models, tumor recurrences were observed in all treated animals. In both the OVCAR3 and OV90 models, tumor recurrences at early time points following CAR T cell treatment were TAG72 low/negative, but maintained MUC16 and/or MUC1 expression. These findings suggest that multi-targeted CAR T cells approaches, which have been developed as either tandem (41, 42) or dual CAR strategies (43, 44) may provide more durable therapeutic responses in tumors with high antigen heterogeneity. Importantly, CAR T cells have already been developed for both MUC16 (40) and MUC1 (45) including a tumor-specific glycoform, Tn-MUC1 (46), and therefore, further exploitation of these targets for treating advanced ovarian cancer is in order. Unexpectedly, we observed TAG72 expression in tumor recurrences at later time points, suggesting that while early resistance mechanisms to CAR T cell therapy may be driven by reduction in tumor antigen density, the absence of CAR T cell selective pressure may have allowed for TAG72 to be re-expressed at later time points. Possible mechanisms include reduced TAG72 expression by downregulation of the enzyme α2,6-sialyl-transferase, transient internalization of TAG72 following exposure to CAR T cells, and pre-existing tumor cells with lower TAG72 that are not targeted by CAR T cells. Similar reductions in tumor antigen density have been observed in leukemia relapses following CAR T cell therapy (47). Additional studies are needed in order to gain a more detailed biological understanding of this observation, which may extend to other tumor antigens including those resulting from aberrant glycosylation. Of note, prior studies have demonstrated that type I and II interferons increase the expression of TAG72 (21, 48), which may also be explored in this setting to increase the tumor antigen density for targeting by CAR T cells.

In the current study, we demonstrated that repeat therapy with TAG72-BBζ CAR T cells increased both maximal therapeutic responses as well as disease control in the OV90 model. While we anticipate that eventual antigen escape-dependent tumor recurrences would have been observed even if repeat treatment continued, it is also plausible that a more optimized CAR T cell with increased persistence may obviate need for repeat therapy. It is noteworthy that our TAG72-CAR T cells also showed significant differences in persistence and expansion in the more aggressive OV90 model, when compared with the OVCAR3 model, suggesting that in addition to antigen escape, other mechanisms may also potentially be limiting the durability of the therapy. For instance, reduced *in vitro* T cell expansion and *in vivo* T cell persistence against OV90 may also be, in part, due to the lower *in vitro* IL-2 production upon TAG72-CAR T cell activation. Therefore, increasing T cell persistence in the solid tumor microenvironment is also imperative, and has been demonstrated recently by several groups engineering CAR T cells with additional supportive cytokines (49–51). However, as recently reported for other advanced tumors (52), improved persistence of T cells within ovarian tumors will be likely be achieved in the context of multi-targeted CAR approaches.

## Author contributions

JM and SP conception and design; JM, AK, W-CC, and SP development of methodology; JM, AK, HL, and MR acquisition of data (provided animals, acquired and managed patients, provided facilities, etc.); JM, AK, HL, MR, W-CC, MC, SF, and SP analysis and interpretation of data (e.g., statistical analysis, biostatistics, computational analysis); JM, AK, MR, W-CC, PY, DC, JS, MC, SF, and SP writing, review, and/or revision of the manuscript; JM, AK, MR, W-CC, PY, DC, JS, SF, and SP administrative, technical, or material support (i.e., reporting or organizing data, constructing databases); SF and SP Study supervision.

### Conflict of interest statement

SP, SF, JM, AK, PY, DC, and JS are listed as co-inventors on a patent on the development of TAG72-CAR T cells, which is owned by City of Hope. The remaining authors declare that the research was conducted in the absence of any commercial or financial relationships that could be construed as a potential conflict of interest.
