# Supplementary material for: Effective Targeting of TAG72+ Peritoneal Ovarian Tumors via Regional Delivery of CAR-Engineered T Cells
Source: Front Immunol. 2018 Nov 19;9:2268. doi: 10.3389/fimmu.2018.02268 (PMC6254427; doi:10.3389/fimmu.2018.02268)
Supplement: Supplementary file 1 [file Data_Sheet_1.PDF]

Supplemental Figure 1

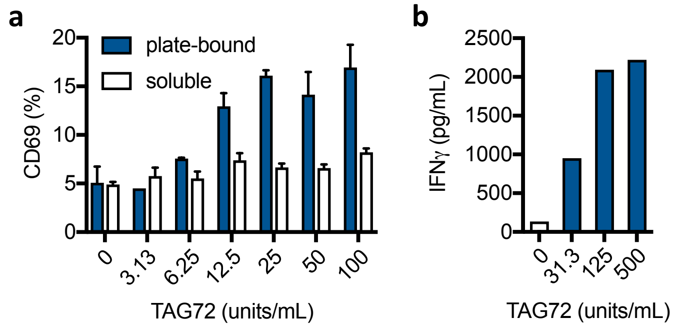

Supplemental Figure 1. TAG72-BBζ CAR T cell activation against purified TAG72 antigen. (a) Activation (expression of CD69) was assessed by flow cytometry with *in vitro* stimulated CAR T cells against soluble or plate-bound purified TAG72 antigen for 24 h at indicated protein amounts (units/mL). (b) IFNγ production by ELISA from TAG72-BBζ CAR T cells against plate-bound purified TAG72 antigen for 48 h at indicated protein amounts (units/mL).

Supplemental Figure 2

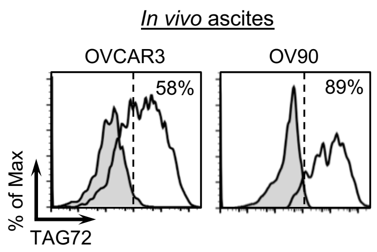

Supplemental Figure 2. TAG72 expression on ascites of tumor-bearing mice. Flow cytometric analysis of TAG72 expression on ascites from OVCAR3 or OV90 tumor-bearing mice.

Supplemental Figure 3

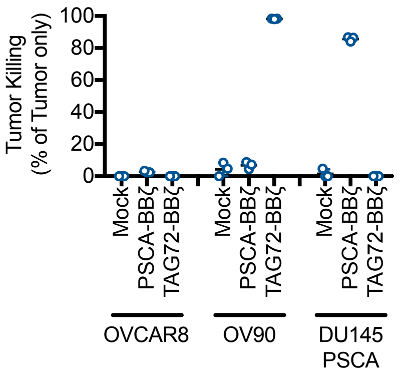

Supplemental Figure 3. Antigen selectivity of TAG72-CAR T cells. Quantification of tumor killing by Mock, TAG72-BBζ or PSCA-BBζ CAR T cells relative to tumor only at an E:T ratio of 1:1, following a 72 h co-culture with antigen-positive and -negative tumor targets as described in Materials and Methods.

Supplemental Figure 4

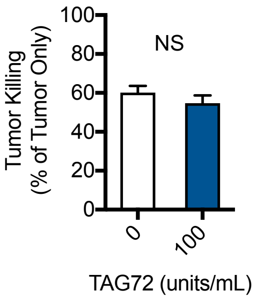

Supplemental Figure 4. Soluble TAG72 antigen does not impact tumor killing of TAG72-BBζ CAR T cells *in vitro*. TAG72-BBζ CAR T cell-mediated tumor killing of OVCAR3 cells in the presence or absence of 100 units/mL of soluble purified TAG72 antigen in a 24 h co-culture assay.

Supplemental Figure 5

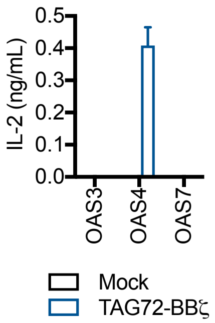

Supplemental Figure 5. IL-2 production by TAG72-BBζ CAR T cells against OAS cells *in vitro*. IL-2 production by Mock and TAG72-BBζ CAR T cells following a 72 h co-culture with freshly thawed OAS cells at an E:T ratio of 1:1.

Supplemental Figure 6

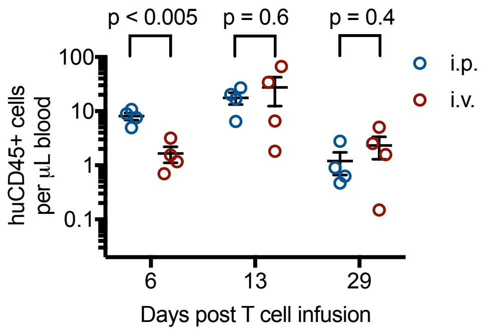

Supplemental Figure 6. Quantification of human CD45+ cells in OVCAR3 model. Quantification of human CD45+ cells per µL blood at 6, 13, and 29 days post treatment. N = 4 per group.

Supplemental Figure 7

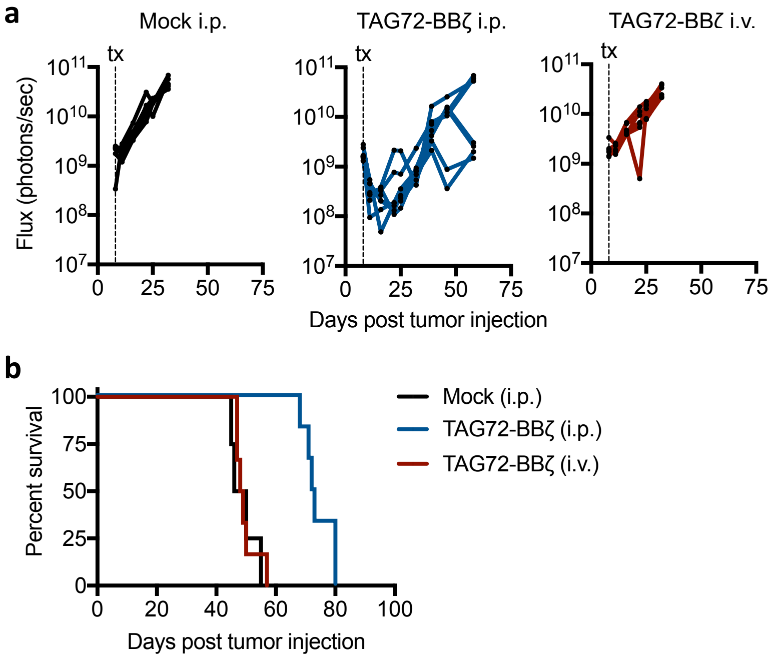

Supplemental Figure 7. TAG72-BBζ CAR T cells delivered i.p. but not i.v. demonstrate anti-tumor activity in OV90 tumor-bearing mice *in vivo*. (a) Quantification of flux (each mouse) from OV90(eGFP/*ffluc*) tumor-bearing mice treated i.v. or i.p. with Mock or TAG72-BBζ CAR T cells. (b) Kaplan-Meier survival for Mock and TAG72-BBζ CAR T cell treated mice. N ≥ 4 mice per group.

Supplemental Figure 8

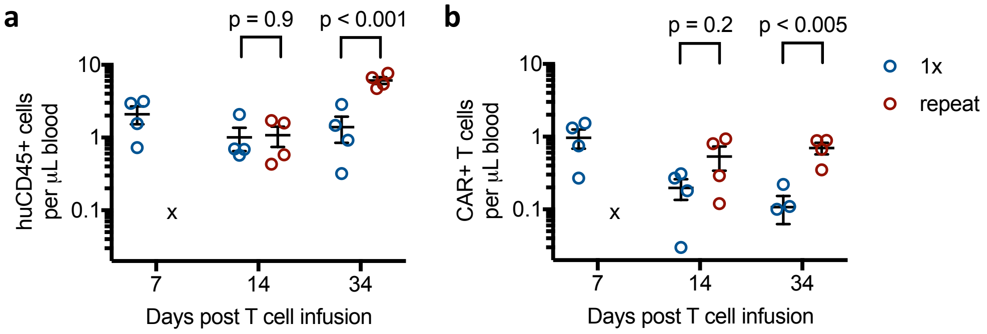

Supplemental Figure 8. Quantification of human CD45+ and CAR+ cells in OV90 model. Quantification of human CD45+ (a) and CAR+ (b) cells per uL blood at 7, 14, and 34 days post treatment. N = 4 per group.
